# An improved, versatile and efficient modular plasmid assembly system for expression analyses of genes in *Xanthomonas oryzae*

**DOI:** 10.1111/mpp.13033

**Published:** 2021-01-24

**Authors:** Lifang Zou, Cuiping Zhang, Yilang Li, Xiaofei Yang, Yanyan Wang, Yichao Yan, Ruihuan Yang, Mengsang Huang, Fazal Haq, Ching‐Hong Yang, Gongyou Chen

**Affiliations:** ^1^ School of Agriculture and Biology Shanghai Jiao Tong University Shanghai China; ^2^ Key Laboratory of Urban Agriculture by Ministry of Agriculture of China Shanghai Jiao Tong University Shanghai China; ^3^ State Key Laboratory of Microbial Metabolism School of Life Sciences & Biotechnology Shanghai Jiao Tong University Shanghai China; ^4^ Department of Biological Sciences University of Wisconsin Milwaukee Wisconsin USA

**Keywords:** modular plasmid assembly, pHM1, promoter‐probe vector, protein expression vector, *Xanthomonas oryzae*

## Abstract

*Xanthomonas oryzae* pathovars *oryzae* (Xoo) and *oryzicola* (Xoc) infect rice, causing bacterial blight and bacterial leaf streak, respectively, which are two economically important bacterial diseases in paddy fields. The interactions of Xoo and Xoc with rice can be used as models for studying fundamental aspects of bacterial pathogenesis and host tissue specificity. However, an improved vector system for gene expression analysis is desired for Xoo and Xoc because some broad host range vectors that can replicate stably in *X*. *oryzae* pathovars are low‐copy number plasmids. To overcome this limitation, we developed a modular plasmid assembly system to transfer the functional DNA modules from the entry vectors into the pHM1‐derived backbone vectors on a high‐copy number basis. We demonstrated the feasibility of our vector system for protein detection, and quantification of virulence gene expression under laboratory conditions and in association with host rice and nonhost tobacco cells. This system also allows execution of a mutant complementation equivalent to the single‐copy chromosomal integration system and tracing of pathogens in rice leaf. Based on this assembly system, we constructed a series of protein expression and promoter‐probe vectors suitable for classical double restriction enzyme cloning. These vector systems enable cloning of all genes or promoters of interest from Xoo and Xoc strains. Our modular assembly system represents a versatile and highly efficient toolkit for gene expression analysis that will accelerate studies on interactions of *X*. *oryzae* with rice.

## Graphical abstract

We developed a modular plasmid assembly system to transfer DNA modules from entry vectors into high‐copy number pHM1‐derived vectors and use it for protein detection, mutant complementation, and tracing pathogens in leaves.

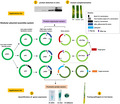

## 1 INTRODUCTION

*Xanthomonas* spp. encompass a broad range of phytopathogenic bacteria that cause diseases in more than 400 different plant hosts, inflicting severe losses on some economically important crops (Timilsina et al., 2020). In rice, *X*. *oryzae* pv. *oryzae* (Xoo) causes bacterial blight by invading the vascular tissue, while another pathovar, *X*. *oryzae* pv. *oryzicola* (Xoc), causes bacterial leaf streak by colonizing the mesophyll tissue (Nino‐Liu et al., 2006). Xoo and Xoc are important as models for understanding fundamental aspects of bacterial interactions with plants. To date, however, the functions of merely a small proportion of genes have been explored. Elucidating the biological functions of the remaining genes in host–pathogen interaction is a challenging task for the *X. oryzae* research community.

Vectors for protein expression and promoter probing are essential molecular tools for gene characterization. Previously, we constructed a protein expression vector pH3‐FLAG that has been successfully applied for type III secretion system (T3SS) protein detection in Xoo (Xu et al., 2019). However, the five unique restriction sites in the multiple cloning sites (MCS) of it are not enough for the cloning of most genes from Xoo and Xoc strains. The promoter‐probe vectors usually have a promoterless reporter gene upstream of the MCS. In addition, one or more transcriptional terminators were positioned upstream of the MCS to reduce the interference caused by readthrough transcription from the backbone vector (Miller et al., 2000). An ideal reporter gene should be sensitive and easily quantified under most laboratory conditions. Among many reporter genes *uidA*, *gfp*, *inaZ*, *lacZ*, and *luxCDABE* operon, *uidA* and *gfp* were commonly used to study the molecular mechanisms implicated in *Xanthomonas–*host cell interactions (Andrade et al., 2014; Han et al., 2008; Uliczka et al., 2011). However, the measured value of green fluorescent protein (GFP) was frequently disturbed by a large amount of extracellular polysaccharide (EPS) and xanthan produced by Xoo cells during the operations of fluorometer (Wang et al., 2015). The ideal vector must be stably maintained and efficiently taken up by the Xoo and Xoc cells. Previously, we checked the stability of some widely used broad host range vectors including pHM1, pPROBE‐NT, pUFR034, and pML123 in the Xoo model strain PXO99^A^ using a *hrpG*‐*uidA* reporter, and found that the *hrpG* promoter β‐glucuronidase (GUS) activity is more stable when the reporter segment is constructed in pHM1 than the other three vectors (Xu et al., 2019). However, the low‐copy number of pHM1 in *Escherichia coli* makes it inconvenient for DNA manipulation. Therefore, cloning and assembly of the functional DNA modules into a pHM1 derivative destination vector are technically challenging.

The main techniques for modular DNA assembly comprise classic restriction enzyme cut‐ligation cloning, Gateway cloning, Golden Gate cloning, Gibson assembly, and Nimble cloning (Yan et al., 2020). Gateway cloning is a site‐specific recombination that was used in the plant field in particular (Binder et al., 2014). However, not only are the commercial reagents for this method very expensive, but the recombination site also leaves a 25‐bp scar sequence (He et al., 2018; Yan et al., 2020). Golden Gate cloning has been applied successfully in the modular assembly of multigene constructs in planta and in the phytopathogenic bacterium *Ralstonia solanacearum*, and also was used to assemble the designer transcription activator‐like effectors (dTALEs) (Engler et al., 2014; Wu et al., 2018). This technique relies on type II restriction enzyme sites such as those of *Bsa*I and *Bpi*I, which are shorter than 7 bp (Binder et al., 2014), and frequently occur within target DNA sequences and some destination vectors suitable for Xoo and Xoc strains, such as pHM1. Gibson assembly cloning has been employed successfully to clone TAL effector genes from Xoo and Xoc strains (Li et al., 2019a). It is a quick and robust technique that enables the assembly of multiple overlapping DNA segments in a thermocycler using a programmed protocol (Li et al., 2019a). The DNA recombination is based on the same homologous ends, indicating that the same terminal sequences (Binder et al., 2014; Silayeva & Barnes, 2018), for instance the four tandem copies of T_1_ terminators (T_1_)_4_, should not be used, as this could result in rearrangement of individual parts. Nimble cloning is a newly developed technique based on Gibson assembly cloning that requires a simple mix of the *Sfi*I restriction enzyme and T5 exonuclease (Yan et al., 2020). Although *Sfi*I is a relatively rare cutting enzyme, it still is present in the important backbone area of pHM1 and is difficult to eliminate.

To overcome the limitations discussed above, we developed a modular plasmid assembly system that comprises three entry vectors and three destination vectors allowing the transfer of functional DNA modules on a high‐copy number basis. We confirmed the utility of this assembly system with several assays in vitro, in host rice and nonhost tobacco tissues. Because this new system is simple, highly efficient, and cost‐effective, it will accelerate gene functional analysis and elucidation of more virulence mechanisms in *Xanthomonas* spp.

## 2 RESULTS

### 2.1 Construction of the modular plasmid assembly system

**FIGURE 1:**
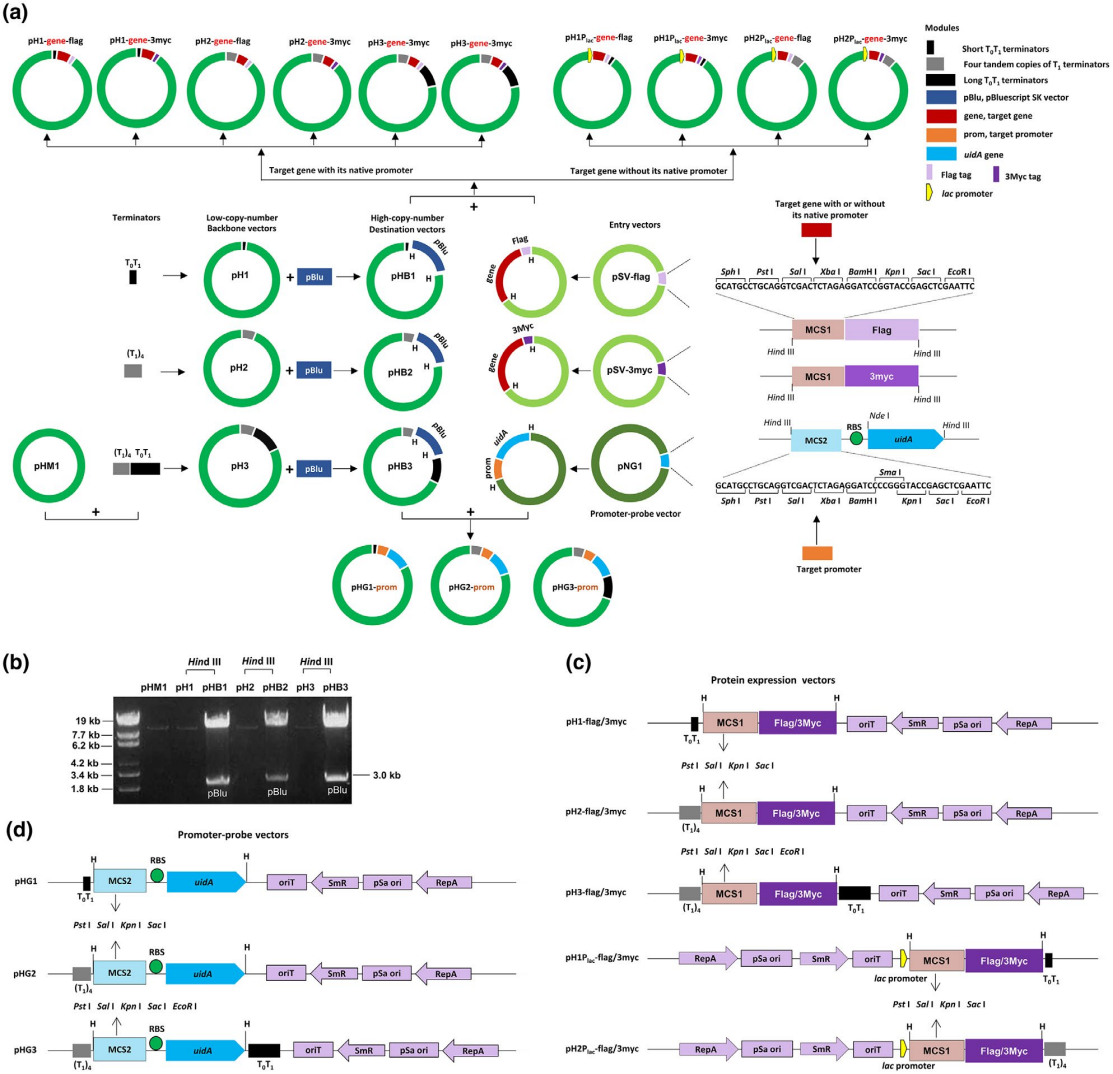
Overview of the modular plasmid assembly system. (a) Schematic diagram of the assembly of the modular plasmids. Red frames indicate a target gene with or without its native promoter. The protein modules in which the target gene carries its native promoter were assembled downstream of the terminators, otherwise they were assembled downstream of the *lac* promoter. Orange frames indicate a target promoter. H, *Hin*dIII enzyme site. (b) Comparison of DNA concentrations between the high‐copy destination vectors and their originating backbone vectors. Twenty microlitres of plasmid DNA from 4 ml of bacterial culture grown overnight was extracted, then digested in a 60‐μl *Hin*dIII enzyme reaction volume. A 5 µl digestion sample was compared by electrophoresis in a 1% agarose gel. (c) Schematic diagram of the protein expression vectors. pH1‐FLAG, pH1‐3Myc, pH2‐FLAG, pH2‐3Myc, pH3‐FLAG, and pH3‐3Myc were designed for gene expression driven by its native promoter, whereas pH1P_lac_‐FLAG, pH1P_lac_‐3Myc, pH2P_lac_‐FLAG, and pH2P_lac_‐3Myc were designed for a constitutive expression driven by the *lac* promoter. (d) Schematic diagram of the promoter‐probe vectors. MCS1 and MCS2 indicate the multiple cloning site of the vectors, and some unique restriction enzyme sites are pointed out. H, *Hin*dIII enzyme site; RBS, ribosome‐binding site; pBlu, the fragment of pBluescript SK vector; T1, transcription terminator T_1_ from the *Escherichia coli rrnB* gene; T_0_, transcription terminator T_0_ from phage λ; oriT, incP origin of transfer; SmR, *aadA* gene encoding spectinomycin resistance; pSa ori, origin of replication from bacterial plasmid pSa; RepA, *trans*‐acting replication function of pSa

The modular plasmid assembly system includes three entry (modular) vectors and three destination vectors. All the destination vectors are variants of pHM1 (Xu et al., 2019), a broad‐range‐host plasmid vector suitable for Xoo and Xoc (Hopkins et al., 1992; Zou et al., 2006). We cloned the T_0_T_1_ and (T_1_)_4_ terminators into pHM1, resulting in two backbone vectors pH1 and pH2, respectively (Figure 1a). Additionally, a cloning vector pBluescript SK with a ColE1 origin of replication was integrated into pH1, pH2, and pH3 (Xu et al., 2019) to increase the copy number of plasmids in *E*. *coli*, resulting in three high‐copy destination vectors pHB1, pHB2, and pHB3 (Figure 1a), which facilitates DNA isolation and manipulation. The DNA concentration of these plasmids is dramatically higher (about 15‐fold) than that of their originating backbone vectors (Figure 1b). Three entry vectors pSV‐FLAG, pSV‐3Myc, and pNG1, are related to the constructions of functional DNA modules. The protein modular vector pSV‐3Myc was created using a similar protocol to that used for the creation of pSV‐FLAG in our previous study (Xu et al., 2019), and the promoter modular vector pNG1 was constructed by replacing the *gfp* gene in pPROBE‐NT with the *uidA* gene from pK18mobGII (Katzen et al., 1999; Miller et al., 2000). Our modular assembly system was performed using the *Hin*dIII‐digested fragments from pHB1, pHB2, and pHB3 plasmids as the recipient backbones. A DNA fragment of interest can be inserted into the entry vectors to create protein or promoter fusion, and subsequently the functional DNA modules can be assembled into the linear recipient backbones by *Hin*dIII cut‐ligation (Figure 1a).

Next, we combined the empty modules of pSV‐FLAG and pSV‐3Myc into the *Hin*dIII sites of pHB1, pHB2, and pHB3 to create a set of protein expression vectors including pH1‐FLAG, pH1‐3Myc, pH2‐FLAG, and pH2‐3Myc that were designed for gene expression driven by its native promoter, as well as pH1P_lac_‐FLAG, pH1P_lac_‐3Myc, pH2P_lac_‐FLAG, and pH2P_lac_‐3Myc that were designed for gene expression without its native promoter but driven by the constitutive *lac* promoter (Figure 1c). These vectors possess the same MCS with four unique restriction sites in the order *Pst*I‐*Sal*I‐*Kpn*I‐*Sac*I, whereas pH3‐3Myc that also was designed for gene expression driven by its native promoter contains the MCS with five unique restriction sites in the order *Pst*I‐*Sal*I‐*Kpn*I‐*Sac*I‐*Eco*RI similar to the previously constructed pH3‐FLAG (Xu et al., 2019). The *uidA* module of pNG1 was assembled into the *Hin*dIII site of pHB1, pHB2, and pHB3, creating a set of promoter‐probe vectors pHG1, pHG2, and pHG3 (Figure 1d). These vectors contain the MCS with four or five unique restriction sites, as well as an optimized ribosome‐binding site (RBS) allowing moderate expression of a gene of interest. This vector system can be used to execute DNA cloning using the classic double enzymes digestion‐ligation strategy and results in a similar construct created by our modular assembly system, but the positive transformants acquired dramatically decreased by about 20‐fold (data not shown).

We mostly used the modular assembly system for the studies described here, whereas we are now also using the new vector system if there is least one naturally occurring *Hin*dIII restriction site in the DNA fragment of interest. According to our experience so far, these two vector systems enable the cloning of almost all genes or promoters of interest from the model strains of Xoo PXO99^A^ and Xoc RS105.

### 2.2 Application for protein detection in vitro

**FIGURE 2:**
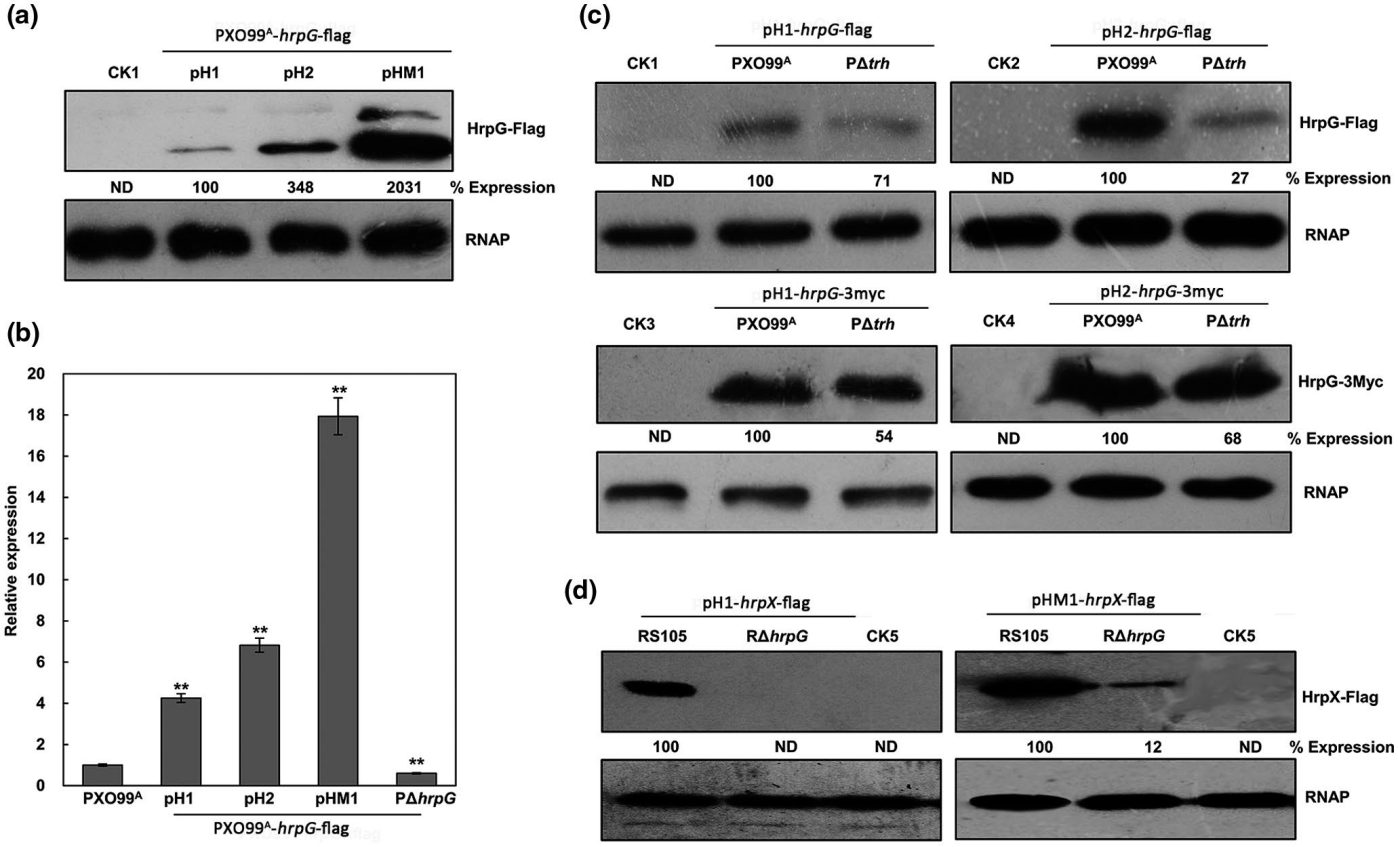
Application of the protein modular assembly system for protein detection in vitro. (a) Graded expression levels of the HrpG‐FLAG proteins were generated by using the T_0_T_1_ and (T_1_)_4_ terminators. (b) Quantitative reverse transcription PCR analysis of *hrpG* mRNAs in the *Xanthomonas oryzae* pv. *oryzae* (Xoo) wild‐type PXO99^A^ carrying the *hrpG*‐FLAG fusion in the pH1, pH2, and pHM1 backbone vectors. The data are mean ± *SD* of the three repeats (**p ≤ .01). Asterisks indicate statistically significant differences in *hrpG* mRNA levels of PXO99^A^ carrying the *hrpG*‐FLAG fusion in the pH1, pH2, and pHM1 backbone vectors compared with that of the wild‐type PXO99^A^. (c) Comparison of HrpG‐FLAG and HrpG‐3Myc expression levels between the wild‐type PXO99^A^ and the *trh* mutant PΔ*trh*. (d) Comparison of the *X*. *oryzae* pv. *oryzicola* (Xoc) HrpX‐FLAG expression levels with or without T_0_T_1_ terminators between the wild‐type RS105 and the *hrpG* mutant RΔ*hrpG*. The total protein extracts were analysed by immunoblotting using anti‐FLAG or anti‐3Myc antibodies. RNAP, RNA polymerase subunit α from *Escherichia coli* was used as a loading control. Value is the relative protein abundance calculated by ImageJ software. Similar results were observed in two independent experiments. ND, not detected. CK1, CK2, CK3, and CK4 indicate the Xoo wild‐type PXO99^A^ carrying an empty vector pH1‐FLAG, pH2‐FLAG, pH1‐3Myc, and pH2‐3Myc, respectively. CK5 indicates the Xoc wild‐type RS105 carrying an empty vector pH1‐FLAG

To confirm the utility of our protein modular assembly system in protein expression detection under laboratory conditions, we chose the representative pathogenicity gene *hrpG* and constructed several Xoo PXO99^A^ strains carrying *hrpG*‐FLAG driven by its native promoter in pH1‐, pH2‐, and pHM1‐based backbone vectors. Our western blotting assays were executed in XOM3, an artificial *hrp*‐inducing medium. The results revealed that a 3‐fold higher expression of the HrpG‐FLAG protein was observed with the pH2 backbone plasmid and a 20‐fold higher expression was observed with the pHM1 plasmid when compared to the pH1‐based construct (Figure 2a), indicating that graded expression levels of HrpG‐FLAG were generated by using the T_0_T_1_ and (T_1_)_4_ terminators. In addition, a quantitative reverse transcription PCR (RT‐qPCR) analysis revealed that about 4‐fold, 7‐fold, and 18‐fold increases of *hrpG* mRNA levels were observed in the Xoo PXO99^A^ strains harbouring the *hrpG*‐FLAG fusion in the pH1, pH2, and pHM1 backbones, respectively, compared to the wild‐type PXO99^A^ (Figure 2b). We obtained similar data after transformation of the *hrpG*‐3Myc fusion cloned in the pH1, pH2, and pHM1 backbones into Xoo PXO99^A^ (data not shown). These results demonstrated that our vector system can be used to customize protein expression levels of the target gene according to experimental needs.

To test the feasibility of this protein modular system in gene regulation analysis, we subsequently transformed the constructs with the *hrpG*‐FLAG and *hrpG*‐3Myc fusions cloned in the pH1 and pH2 backbones into the *trh* mutant PΔ*trh*. The resulting PΔ*trh* strains exhibited significantly reduced HrpG‐FLAG and HrpG‐3Myc expression levels when compared to the wild‐type PXO99^A^ (Figure 2c), which is in agreement with the previous findings that Trh is a positive regulator of the expression of *hrpG* in Xoo (Tsuge et al., 2006; Wang et al., 2016). Similarly, we constructed Xoc with a *hrpX*‐FLAG fusion driven by its native promoter in the pH1 and pHM1 backbones. As previously demonstrated in Xoc, HrpG positively regulates the expression of *hrpX* (Li et al., 2011; Zou et al., 2006). Our western blotting showed that the HrpX‐FLAG expression levels in the *hrpG* mutant RΔ*hrpG* were dramatically lower than that in the Xoc wild‐type RS105, but the HrpX‐FLAG expression levels in RΔ*hrpG* were not detectable when the *hrpX*‐FLAG fusion was cloned in the pH1‐based backbone (containing the T_0_T_1_ terminators) rather than in pHM1 (containing no terminators) (Figure 2d). This suggested that the terminators could effectively eliminate the effect of readthrough transcription from the native backbone vector, which is important under the XOM3 growth medium conditions. Together, these results demonstrate that our protein vector system is an effective tool suitable for protein expression assays in vitro.

### 2.3 Application in mutant complementation

**FIGURE 3:**
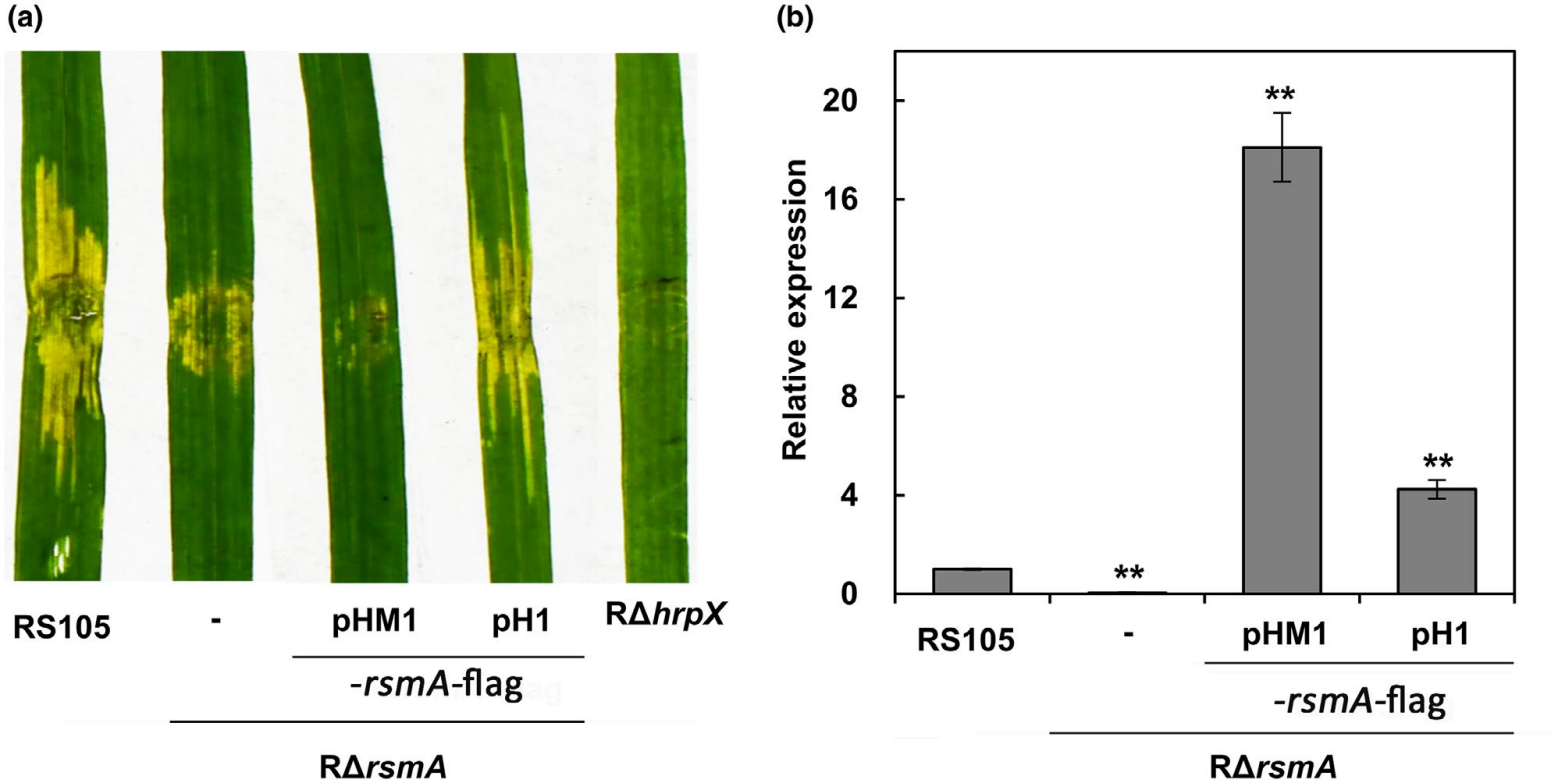
Application of the pH1‐based cloning system in mutant complementation. (a) Water‐soaked symptoms caused by the *Xanthomonas oryzae* pv. *oryzicola* (Xoc) *rsmA* mutant RΔ*rsmA* and its complementary strains. (c) Expression ratios of *rsmA* mRNAs in the *rsmA* complementary strains versus the wildtype RS105. Error bars indicate standard deviation (*SD*). Asterisks indicate statistically significant differences (mean ± *SD*, *n* = 3, **p ≤ .01). The pHM1‐*rsmA*‐FLAG and pH1‐*rsmA*‐FLAG indicate the RS105 *rsmA*‐FLAG fusion with its native promoter cloned in pHM1 and pH1, respectively

Some previous reports described that a chromosomal insertion of a cloned gene based on the mini‐Tn*7* single‐copy system could revert its virulence‐deficient mutant to wild‐type levels; however, overexpression of the gene based on a cloning vector could not (Zou et al., 2012). Our previous report described that a Xoc RS105 *rsmA*‐FLAG fusion with its native promoter cloned in pHM1 could not complement the virulence‐deficient phenotype of the *rsmA* deletion mutant RΔ*rsmA* (Song et al., 2017) (Figures 3a and S1a). To test whether our pH1‐based expression system could exert gene complementation, we cloned the same *rsmA*‐FLAG fusion downstream of the T_0_T_1_ terminators in the pH1 backbone vector. Rice inoculation revealed that expression of the *rsmA*‐FLAG fusion with pH1 in RΔ*rsmA* was capable of restoring virulence to the wild‐type levels (Figures 3a and S1a). In addition, the RT‐qPCR analysis revealed that RΔ*rsmA* with pH1‐*rsmA*‐FLAG only exhibited a 4‐fold increase of the *rsmA* mRNA levels relative to the wild type, indicating that about 4.5‐fold reduction of the *rsmA* mRNA levels was observed in RΔ*rsmA* with pH1‐*rsmA*‐FLAG compared to the one with pHM1‐*rsmA*‐FLAG (Figure 3b). We transformed all constructs with the *hrpG*‐FLAG and *hrpG*‐3Myc fusions into the Xoo *hrpG* mutant PΔ*hrpG* and found that all complementary strains restored pathogenicity on rice (Figure S1b). The results indicate that our protein modules are adequate for gene functional complementation and the pH1‐based cloning strategy can be used as an alternative to the mini‐Tn*7*‐based chromosomal insertion in gene functional complementation.

### 2.4 Application for quantification of T3SS gene expression in vitro

**FIGURE 4:**
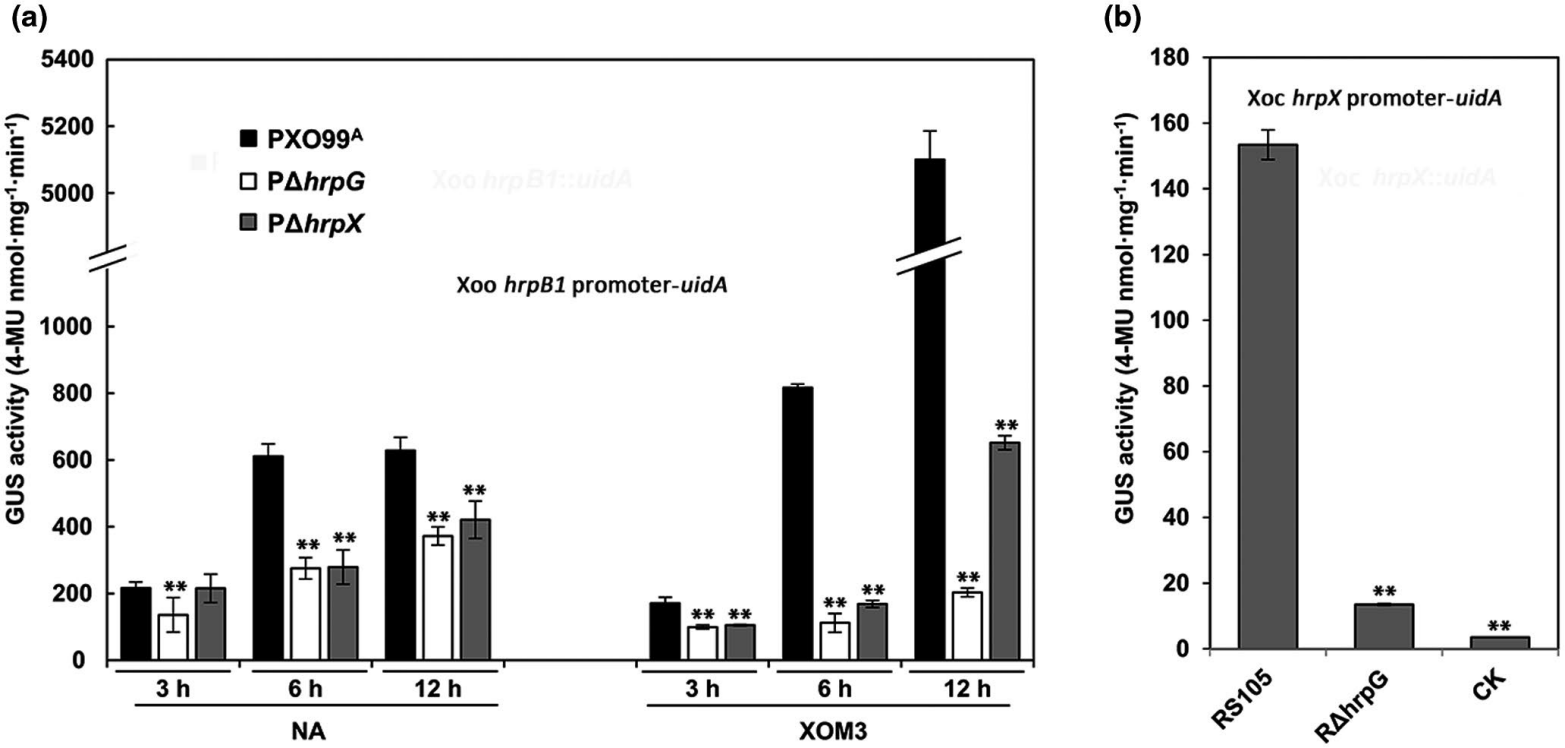
Application of the promoter modular assembly system for quantifying T3SS gene expression in vitro. (a) The *hrpB1* promoter β‐glucuronidase (GUS) activities of the *Xanthomonas oryzae* pv. *oryzae* (Xoo) wild‐type PXO99^A^, PΔ*hrpG*, and PΔ*hrpX* induced by nutrient‐rich (NA) and XOM3 media at 3, 6 and 12 hr after induction. (b) The *hrpX* promoter GUS activities of the *X*. *oryzae* pv. *oryzicola* (Xoc) wild‐type RS105 and the *hrpG* mutant RΔ*hrpG*. Error bars indicate standard deviation (*SD*). Asterisks indicate statistically significant differences (mean ± *SD*, *n* = 3, **p ≤ .01). Similar results were observed in three individual experiments, and the representative result is shown here. CK indicates the Xoc wild‐type RS105 carrying an empty vector control

To confirm the utility of our modular assembly system in promoter activity analysis in vitro, we chose the representative T3SS gene *hrpB1*, which is the first gene in the *hrpB* operon of the genomic core *hrp* cluster of Xoo (Zou et al., 2006), and constructed the *hrpB1* promoter‐*uidA* reporter fusion in the pH3 backbone. The quantitative GUS assays showed that the *hrpB1* promoter GUS activity of the wild‐type PXO99^A^ was dramatically higher in XOM3 medium than in NB (NA broth, a nutrient‐rich medium) at 6 and 12 hr after induction (hai) (Figure 4a), indicating that *hrp* gene expression is significantly increased from 6 to 12 hai. Meanwhile, the *hrpB1* promoter GUS activity was significantly lower in the *hrpG* mutant PΔ*hrpG* and the *hrpX* mutant PΔ*hrpX* than that in the wild type at 6 or 12 hai either in XOM3 or in NB, which is in agreement with the result that the expression of T3SS genes in Xoo depends on HrpX and HrpG (Wang et al., 2016). In addition, we obtained data for the application of these vectors in the analysis of Xoc *hrpX* expression in the Xoc *hrpG* mutant RΔ*hrpG* (Figure 4b). These results suggest that our *uidA* promoter modular assembly system is adequate for quantifying T3SS gene expression of Xoo and Xoc in vitro.

### 2.5 Application for analyses of T3SS gene expression on host cell contact

**FIGURE 5:**
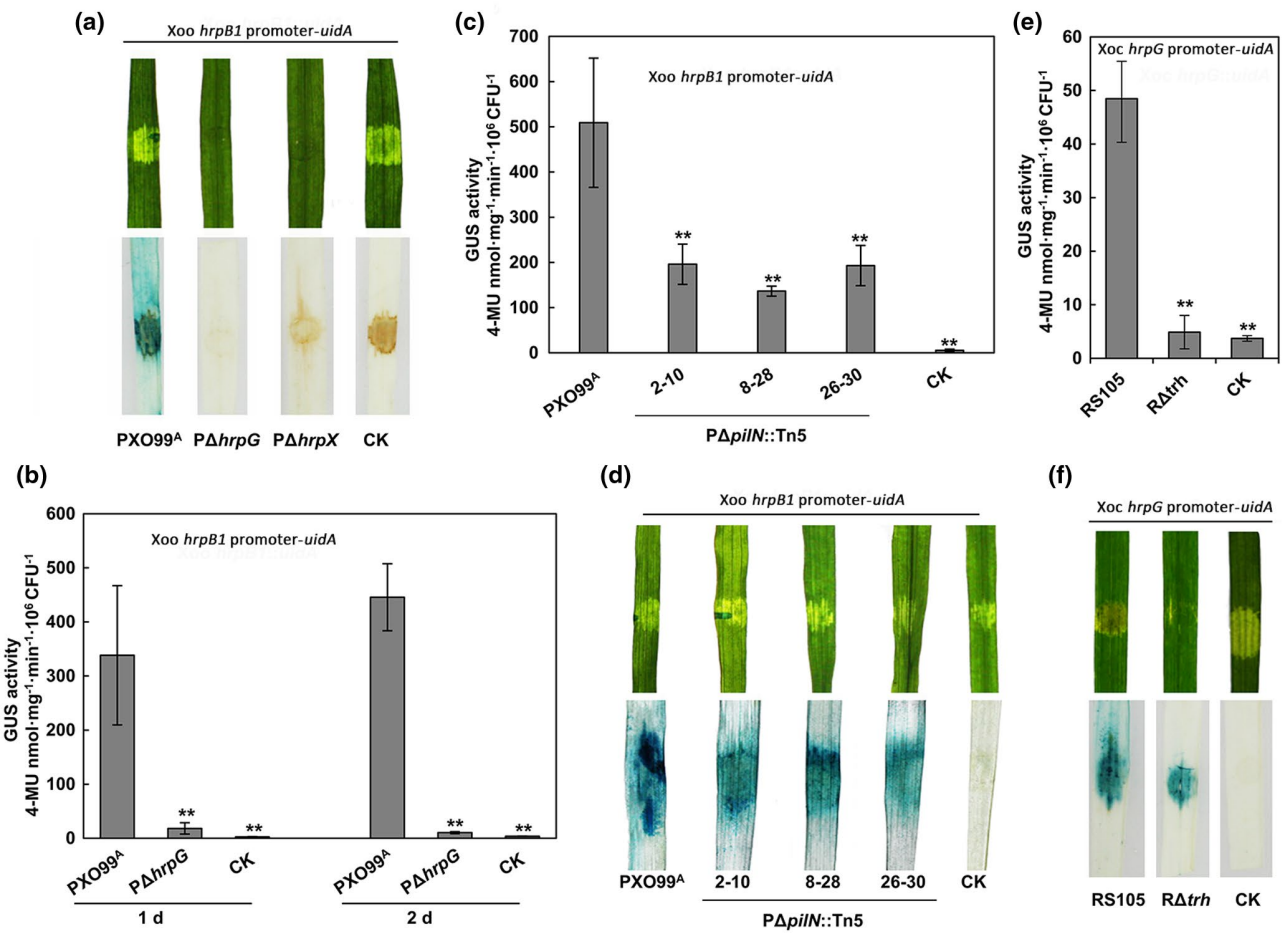
Application of the promoter modular assembly system for analyses of type III secretion system (T3SS) gene expression on host cell contact. (a) Qualitative β‐glucuronidase (GUS) histochemical staining of the *Xanthomonas oryzae* pv. *oryzae* (Xoo) wild‐type PXO99^A^, PΔ*hrpG*, and PΔ*hrpX* harbouring pHG3‐*hrpB1*. (b) Quantification of *hrpB1* expression of the wild‐type PXO99^A^ and PΔ*hrpG* in the early stage of infection. (c) Quantification and (d) qualitative GUS analyses for the *hrpB1* promoter of the Xoo wild‐type PXO99^A^ and three *pilN* mutants in rice leaves. 2–10, 8–28, and 26–30 are Tn*5* insertion mutants of the *pilN* gene in the PXO99^A^ background. (e) Quantification and (f) qualitative GUS analyses for the *hrpG* promoter of the *X*. *oryzae* pv. *oryzicola* (Xoc) wild‐type RS105 and the *trh* mutant RΔ*trh* in rice leaves. Bacterial suspensions (OD_600_ = 2.0) were inoculated using a needleless syringe into the leaves of a 2‐week‐old rice line IR24. The infected leaves at 1 or 2 days postinoculation (dpi) were collected for GUS activity analyses and GUS staining. Bars indicate standard deviation (*SD*) and asterisks indicate statistically significant differences (mean ± *SD*, *n* = 3, **p ≤ .01). CK indicates the Xoo wild‐type PXO99^A^ and the Xoc wild‐type RS105 carrying an empty vector control

To check whether our promoter‐probe vectors work well in Xoo in association with rice cells, we first performed a qualitative GUS histochemical staining assay. The result showed that PΔ*hrpG* and PΔ*hrpX* harbouring the *hrpB1* promoter‐*uidA* fusion exhibited undetectable GUS activity for *hrpB1* promoter in leaves of IR24, a rice line susceptible to Xoo, at 3 days postinoculation (dpi) (Figure 5a). To precisely quantify T3SS gene expression in the early stage of infection, we combined the data of bacterial populations of the wild‐type PXO99^A^ and PΔ*hrpG* in IR24 at 0, 1, 2, and 3 dpi (Figure S2a), which was neglected in the qualitative GUS assay. The quantitative GUS analysis showed that the *hrpB1* promoter GUS activity per 10^6^ bacterial cells was significantly reduced in PΔ*hrpG* compared to the wild‐type PXO99^A^ at 1 and 2 dpi (Figure 5b), which is a more obvious result than the one observed in vitro in XOM3 medium (Figure 4a).

Furthermore, we checked the application of this method for quantifying *hrpB1* expression in three Xoo *pilN* mutants, 2–10, 8–28, and 26–30, that exhibited impaired virulence in IR24 in our previous study (Li et al., 2020). We found that all of them significantly reduced the *hrpB1* promoter GUS activity when compared to the wild‐type PXO99^A^ in rice tissues at 2 dpi (Figure 5c), which is in agreement with the result from the qualitative GUS histochemical staining (Figure 5d) and in XOM3 medium (Figure S2b). Data were also acquired when the Xoc wild‐type RS105 and the *trh* mutant RΔ*trh* carrying a *hrpG* promoter‐*uidA* fusion were inoculated into the leaf of IR24 (Figure 5e,f). These experiments demonstrated that our *uidA* vector system is appropriate for quantitative analyses of T3SS gene expression during the initial phase of Xoo and Xoc interacting with host rice cells.

### 2.6 Application for monitoring T3SS gene expression on nonhost cell contact

**FIGURE 6:**
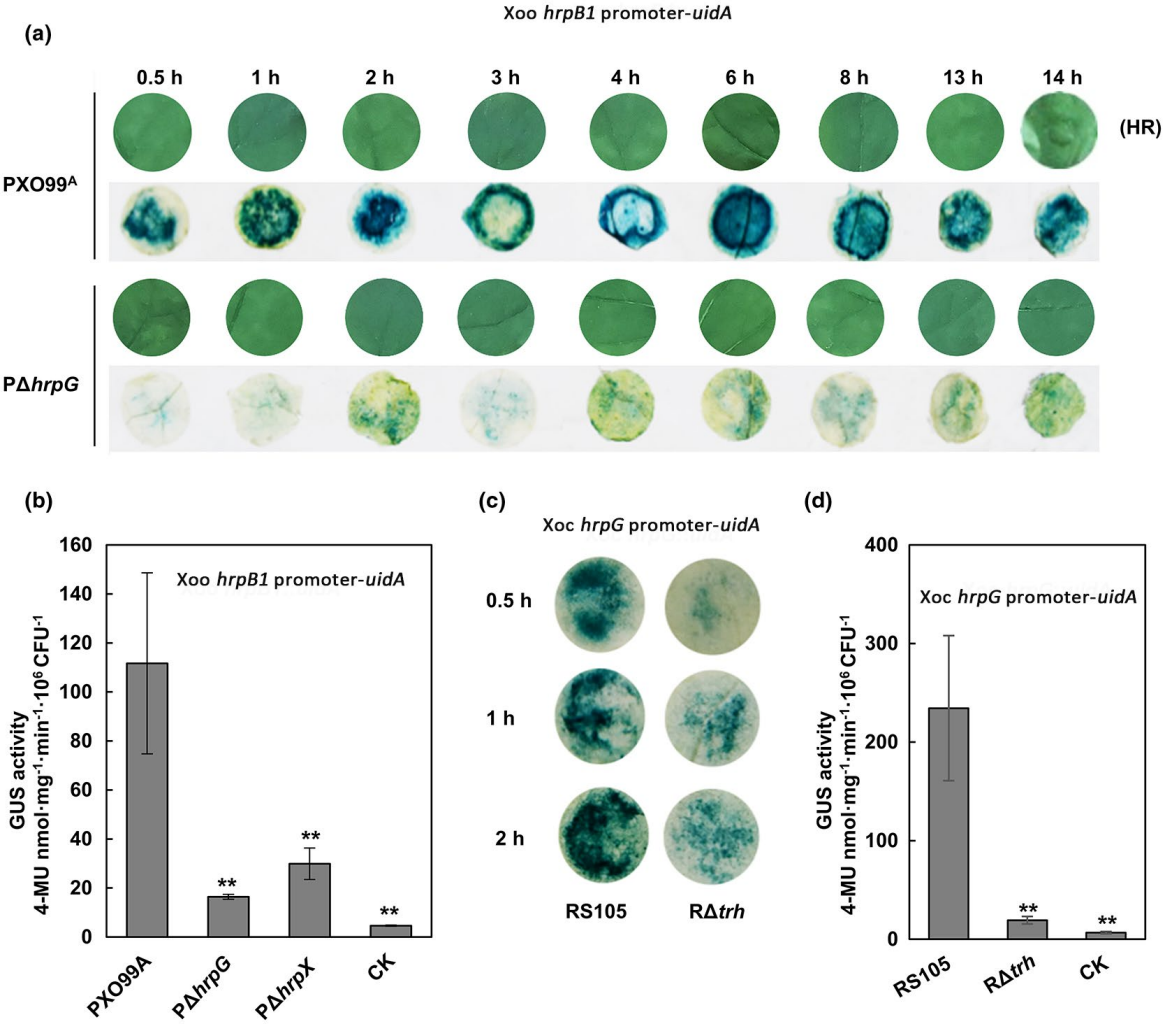
Application of the promoter modular assembly system for monitoring and quantifying type III secretion system (T3SS) gene expression upon nonhost cell contact. (a) Monitoring *hrpB1* expression of the *Xanthomonas oryzae* pv. *oryzae* (Xoo) wild‐type PXO99^A^ and the *hrpG* mutant PΔ*hrpG* upon nonhost cell contact. (b) The *hrpB1* promoter β‐glucuronidase (GUS) activities of the Xoo wild‐type PXO99^A^, PΔ*hrpG*, and PΔ*hrpX* per 10^6^ cfu cells upon nonhost tobacco contact at 2 hr postinoculation. (c) Quantitative and (d) quantitative GUS analyses for the *hrpG* promoter of the *X*. *oryzae* pv. *oryzicola* (Xoc) wild‐type RS105 and RΔ*trh* upon nonhost tobacco contact. Hypersensitive response (HR) observation (upper line) and the corresponding GUS staining for *hrpB1* promoter GUS activity (below line) were indicated in (a). Bars indicate standard deviation (*SD*) and asterisks indicate statistically significant differences (mean ± *SD*, *n* = 3, **p ≤ .01). CK indicates the Xoo wild‐type PXO99^A^ and the Xoc wild‐type RS105 carrying an empty vector control

T3SS genes are essential for Xoo and Xoc to trigger a hypersensitive response (HR) on the nonhost tobacco, which is observed at 14 hr postinoculation (hpi) (Figure 6a), while T3SS gene expression is inducing by stimuli derived from tobacco. To confirm the efficacy of our vectors for assessing T3SS gene expression on nonhost cell contact, we first analysed the expression pattern of *hrpB1* during Xoo infection at 0.5, 1, 2, 3, 4, 6, 8, 13, and 14 hpi by GUS histochemical staining. The results showed that *hrpB1* was up‐regulated in the wild‐type PXO99^A^ during the early stage of Xoo infection, reaching the highest expression level at 6 hpi, then was down‐regulated gradually (Figure 6a). Consistent with this, *hrpB1* was also up‐regulated in PΔ*hrpG* at the early stage of infection (Figure 6a), indicating that the first 6 hr of Xoo infection is critical for T3SS induction analysis. Bacterial growth curves revealed that populations of the wild type and PΔ*hrpG* in tobacco were nearly identical at 4 hpi (Figure S2c). The GUS quantification showed that the *hrpB1* promoter GUS activity was significantly reduced in PΔ*hrpG* and PΔ*hrpX* compared to that in the wild type at 2 hpi (Figure 6b), which was consistent with the result obtained by GUS histochemical staining. The expected result was obtained when the Xoc *hrpG* promoter was tested in the *trh* mutant RΔ*trh* (Figure 6c,d). These results indicate that the *uidA* reporter vectors worked properly in tobacco, and the methods we developed can be used in rapid quantification of T3SS gene expression during the first few hours of Xoo and Xoc interacting with nonhost plants.

### 2.7 Application for tracing pathogens in rice leaves

**FIGURE 7:**
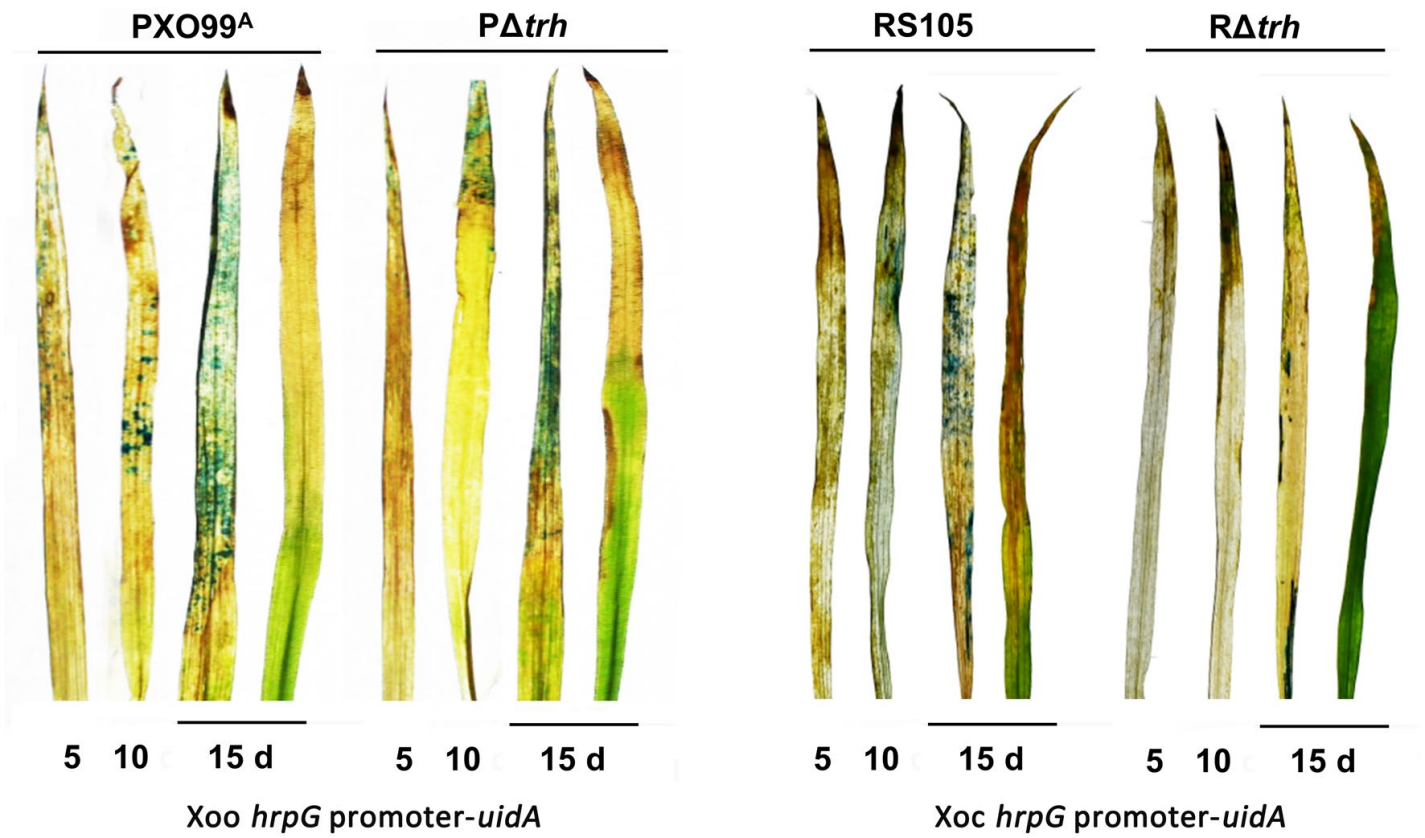
Observation of the pathogenesis caused by the *Xanthomonas oryzae* pv. *oryzae* (Xoo) and *X*. *oryzae* pv. *oryzicola* (Xoc) *uidA* tracing strains. Bacterial suspensions (OD_600_ = 1.0) of the Xoo wild‐type PXO99^A^ and its *trh* mutant PΔ*trh* as well as the Xoc wild‐type RS105 and its *trh* mutant expressing a *hrpG*‐*uidA* fusion were sprayed on the leaves of 4‐week‐old IR24 rice leaves. The infected leaves at 5, 10, and 15 days postinoculation (dpi) were stained in a β‐glucuronidase (GUS) staining solution, then discoloured by 70% ethanol. The symptoms of bacterial leaf blight (leaf on right in the 15 d pair) were photographed at 15 dpi

Xoo invades rice vascular tissue through hydathodes at the leaf tip and leaf margin, whereas Xoc penetrates the mesophyll parenchyma through stomata (Nino‐Liu et al., 2006). We previously found that strains expressing the *uidA* gene under the control of a *hrp* promoter were easily traceable in the vascular system after injection inoculation (Li et al., 2019b). To trace the infection process of Xoo and Xoc in rice, we sprayed a bacterial suspension on rice leaves of line IR24, mimicking natural infection. At the early stages of infection, no obvious water‐soaked symptoms were observed in rice leaves at 5 dpi; however, inoculation with the Xoo wild‐type PXO99^A^ and Xoc wild‐type RS105 containing a *hrpG* promoter‐*uidA* cassette caused scattered blue staining of rice leaves. Disease symptoms were observed at 10 and 15 dpi. Meanwhile, strong GUS staining could be observed at the leaf tip infected by PXO99A and in the central area of the leaf infected by RS105 (Figure 7), indicating that the changes of localization and propagation of Xoo and Xoc in rice tissues were successfully detected. By contrast, the *trh* mutants PΔ*trh* and RΔ*trh* with the *hrpG* promoter‐*uidA* reporter exhibited less GUS staining compared with their corresponding wild‐type strain, suggesting that spread of the *trh* mutants significantly decreased in rice tissues, which is in agreement with the previous study (Tsuge et al., 2006; Wang et al., 2016). These results indicate that our *uidA* reporter system can effectively trace infection of Xoo and Xoc and monitor the bacterial population in a rice leaf, as well as providing an efficient method for evaluating the contribution of some functional genes on the overall pathogenesis.

## 3 DISCUSSION

To establish a highly efficient experimental platform for gene expression analysis in *X*. *oryzae*, we developed a modular plasmid assembly system. We tested this system with several crucial virulence genes (such as T3SS genes) under laboratory conditions, and established a standardized procedure to quantify gene expression during the initial phase of *X*. *oryzae* interacting with host rice and nonhost tobacco. We demonstrated the usefulness of our system for gene expression analysis at both the transcriptional and translational levels. This system will provide robust technical support in studies of *X*. *oryzae–*rice interactions.

Because of the genetically stable replication and delivery efficiency, pHM1 is the most widely used cloning vector in *X*. *oryzae*, especially in the model strain Xoo PXO99^A^ (Hopkins et al., 1992). However, a good expression system based on it is lacking because of its low copy number in *E*. *coli*. Our modular assembly system overcomes this limitation, and all functional modules are transferred between high‐copy‐number plasmids. Although the destination vectors can produce the same sticky ends as their former backbones when digested by *Hin*dIII, the DNA yields are dramatically increased by about 15‐fold, greatly improving cloning efficiency and positive transformants acquired. Currently, a similar assembly method has been reported and used to boost the expression levels of TAL effector genes from *X*. *oryzae* in rice (Li et al., 2019a). In this case, to facilitate DNA isolation and manipulation, the recipient vector pHM1‐Gib was integrated into the ColE1 origin of replication from pBluescript in the Gibson assembly processing (Li et al., 2019a).

The modified pUC18 polylinker with more than eight restriction sites in three entry vectors allows for the cloning of more coding or promoter sequences. Although the transfer of functional modules between vectors depends on a *Hin*dIII digestion‐ligation strategy, the *Hin*dIII recognition sites occur at low frequencies, with only 60 and 55 sites per million base pairs in the Xoo PXO99^A^ and Xoc RS105 genomes, respectively, which is in contrast to the 146 and 154 *Sfi*I sites per million base pairs. There is one *Sfi*I recognition site in the important backbone area of pHM1 and it is difficult to eliminate it. This indicates that our modular assembly system has some clear advantages over the current Nimble cloning system for DNA constructs based on the Xoo PXO99^A^ and Xoc RS105 genomes (Yan et al., 2020). Our protein expression and promoter‐probe vectors system can also be used when there is at least one naturally occurring *Hin*dIII site in the DNA fragment of interest. It allows for the cloning of fragments through a classic double digestion‐ligation strategy in one‐step cloning. The probability of coexistence of a *Hin*dIII and other four or five unique enzyme (in the MCS of our vector system) recognition sites in a DNA segment of interest is very rare. This indicates that our cloning system is likely to be sufficient for cloning almost all genes from the model strains Xoo PXO99^A^ and Xoc RS105. Furthermore, our assembly system and the relevant vectors are applicable for other important xanthomonads, such as *X*. *campestris* pv. *campestris* and *X. citri* subsp*. citri*, where the use of the pHM1‐derived plasmids has been reported in prior studies (Chengid et al., 2019; Pan et al., 2018; Zhou et al., 2018).

Most existing bacterial expression systems are derivatives of the pET vector series, in which the terminator cassette is usually positioned downstream of the epitope tag (Dammeyer et al., 2013; Prior et al., 2010). A great improvement in our protein modular assembly system is that the T_0_T_1_ and (T_1_)_4_ terminators were positioned upstream of the MCS‐tag cassette, which endows this system with more functionality. First, our system is very effective for protein detection in the study of gene regulation. The T_0_T_1_ and (T_1_)_4_ terminators not only avoid readthrough from the vector background, increasing the sensitivity of the protein tags, but also confer graded expression levels of the target gene, allowing customization of the target protein with relative expression levels according to experimental needs. Secondly, the pH1‐based cloning system could execute a substitution of the mini‐Tn*7*‐based single‐copy cloning system in gene functional complementation. The prior study estimated the copy number of pSa replicons (origin of replication of pHM1) to be 2 to 3 in *E*. *coli* (Innes et al., 1988), which is consistent with our result that a cloned gene in the pH1 vector backbone could maintain about 3‐fold higher expression level relative to its single copy in the wild type. Therefore, the expression of *rsmA* based on pH1 could recover its virulence‐deficient mutant RΔ*rsmA* to wild‐type levels, but its expression based on the pHM1 backbone could not (Song et al., 2017). We speculate that this was due to overexpression of *rsmA* in the pHM1‐based background, and indicates that RsmA might execute a subtle regulatory network in Xoc. A similar study reported that a chromosomal insertion of *slyA* in *Dickeya dadantii* 3937 based on the mini‐Tn*7* single‐copy system could return the enhanced *hrpS* expression of its mutant to wild‐type levels, but its expression based on the cloning vector pML123 could not (Zou et al., 2012). Although the mini‐Tn*7* system has been successfully applied for single‐copy gene complementation in several bacterial strains, including *Xanthomonas* spp. (Figueroa‐Cuilan et al., 2016; Jittawuttipoka et al., 2009; Kumar et al., 2010; Liu et al., 2014), the transposition success rate of the mini‐Tn*7* elements into the Xoo PXO99^A^ and Xoc RS105 genome was less than expected (unpublished data). In addition, the relative vector constructions, verification of transposition events, and marker excision are time‐consuming and laborious.

The *X. oryzae–*rice pathosystem depends on a T3SS encoded by a *hrp* gene cluster (called T3SS genes) to inject T3SS effectors into rice cells for disease development (Zou et al., 2011). We found that T3SS gene expression was significantly enhanced with induction time between 6 and 12 hr in XOM3, therefore we optimized the measuring method, changing the inducing time of bacterial cells to 6 hr instead of the previous 3 hr. Although XOM3 is a nutrient‐poor artificial medium, it cannot accurately mimic infection scenarios in the host rice. There is increasing evidence that there are significant differences between the regulation patterns of some virulence genes in the *hrp*‐inducing medium and in the host environment (Zhou et al., 2017). Thus, quantification of T3SS gene expression in rice tissues is a critical technique to decipher the functions of the T3SS genes in *X*. *oryzae* pathogenesis. Experiments by growth in XOM3 are generally performed with the same density of bacteria, but this is hard to regulate when bacteria enter rice tissues, especially in the case of bacteria with loss of pathogenicity. Our method is suitable for quantitative analysis of T3SS gene expression of *X. oryzae* on host cell contact. This technical advance will accelerate our understanding of biological events in Xoo–rice interaction, and it is also important to address gene function analyses. Our *uidA* reporter system also allows for quantification of T3SS gene expression on nonhost cell contact. First, we demonstrated that T3SS gene expression is highly up‐regulated soon after inoculation with Xoo, which is consistent with previous reports (Cui et al., 2018). Secondly, we defined that 2 hpi was the ideal timing for quantifying T3SS gene expression in tobacco leaves. This nonhost interaction system is hardly ever used by researchers to elucidate the virulence regulation networks in *X. oryzae*, but it is highly efficient and time‐saving.

In summary, we have established a cloning system by a modular assembly that effectively overcomes the low efficiency of DNA genetic manipulation of low‐copy‐number vectors. This system is simple, cost‐effective, versatile, and efficient, and is suitable for gene expression analysis in *X*. *oryzae* strains and in other *Xanthomonas* species.

## 4 EXPERIMENTAL PROCEDURES

### 4.1 Bacterial strains, plasmids, plants, and growth conditions

The Xoo and Xoc strains were grown at 28 °C in a nutrient‐rich agar (NA) medium or NA broth (NB) medium (Xu et al., 2019). *E. coli* strains were cultured at 37 °C in Luria‐Bertani (LB) medium. XOM3 is an inducing medium for *hrp* genes from Xoc and Xoo strains (Xiao et al., 2007). All bacterial strains and plasmids used in this study are listed in Table S1.

### 4.2 DNA manipulation and plasmid construction

All DNA fragments in this study were amplified using PXO99^A^ (GenBank: NC_010717) and RS105 (GenBank: CP011961) genomic DNA as a template. The primers were synthesized by Sangon Biotech Co., Ltd and are listed in Table S2. The enzymes involved in molecular manipulation were purchased from TaKaRa Biomedical Technology Co., Ltd. DNA extraction and gel purification were conducted as the standard protocols of GBS Biotechnology.

#### 4.2.1 Construction of the entry vectors

The complete MCS‐3Myc DNA cassette was synthesized, then ligated into the *Eco*RV site of the pUC57‐simple vector by OGENE BIOTEK, creating pSV‐3Myc. The fragment containing the *uidA* gene amplified from pK18mobGII using primers gusA‐*F*(NdeI) and gusA‐R(NdeI) was ligated into the *Nde*I site of pPROBE‐*gfp*, resulting in pNG. The MCS‐*uidA* DNA cassette of pNG was amplified using primers gusA‐F and gusA‐R, then was cloned into the *Hin*dIII site of pNG to create pNG1. The complete nucleotide sequence of pNG1 (accession number MK836322) has been deposited in GenBank.

#### 4.2.2 Construction of the destination vectors

The DNA fragment of the T_0_T_1_ terminators from plasmid pUC18T‐mini‐Tn*7*T‐Gm (GenBank: AY599232) was amplified using primers T_0_1‐F and T_0_1‐R, then was cloned into pHM1 (GenBank: EF059993) via *Eco*RI and *Hin*dIII to create pH1. Similarly, the fragment containing four T_1_ terminators from pPROBE‐*gfp* was amplified using primers T_1_4‐F and T_1_4‐R, then was ligated into pHM1 via *Eco*RI and *Hin*dIII to create pH2. The pH1, pH2, and pH3 (GenBank: MH445408) were integrated into the cloning vector pBluescript SK, resulting in pHB1, pHB2, and pHB3.

#### 4.2.3 Construction of the protein expression vectors

The MCS‐FLAG/3Myc cassettes of pSV‐FLAG and pSV‐3Myc were ligated into pHB1, pHB2, and pHB3 *Hin*dIII, respectively, to create a set of protein expression vectors. The cassettes were positioned downstream of the T_0_T_1_ or (T_1_)_4_ terminators in pH1 and pH2 to generate pH1‐FLAG/3Myc and pH2‐FLAG/3Myc, and were positioned downstream of the *lac* promoter in pH1 and pH2, resulting in pH1P_lac_‐FLAG/3Myc and pH2P_lac_‐FLAG/3Myc. The MCS‐3Myc cassette was inserted into pH3, resulting in pH3‐3Myc. pH1‐FLAG/3Myc, pH2‐FLAG/3Myc, and pH3‐3Myc were designed for gene expression with its native promoter, whereas pH1P_lac_‐FLAG/3Myc and pH2P_lac_‐FLAG/3Myc were designed for gene expression driven by a constitutive *lac* promoter.

The fragment containing the Xoo *hrpG* open reading frame and its native promoter was amplified from PXO99^A^ using primers *hrpG*‐F and *hrpG*‐R, then was cloned via *Sal*I and *Kpn*I into pSV‐FLAG to create pSV‐*hrpG*‐FLAG. The recombinant *hrpG*‐FLAG was ligated into pHB1 and pHB2 to create pH1‐*hrpG*‐FLAG and pH2‐*hrpG*‐FLAG, respectively. The same *hrpG* functional fragment was cloned into pSV‐3Myc to create pSV‐*hrpG*‐3Myc. The recombinant *hrpG*‐3Myc was ligated into pHB1 and pHB2 downstream of the T_0_T_1_ and (T_1_)_4_ terminators, resulting in pH1‐*hrpG*‐3Myc and pH2‐*hrpG*‐3Myc, respectively. Similarly, the fragment containing the *hrpG* open reading frame without its native promoter was amplified using *hrpG* ORF‐F and *hrpG*‐R, then was cloned into pSV‐FLAG to create pH1p_lac_‐*hrpG*‐FLAG and pH2p_lac_‐*hrpG*‐FLAG. Following the same procedure, the plasmids pH1‐*hrpXoc*‐FLAG and pHM1‐*hrpXoc*‐FLAG were constructed using the primers hrpXoc‐F and hrpXoc‐R.

#### 4.2.4 Construction of the promoter‐probe vectors

The MCS‐*uidA* cassette of pNG1 was positioned downstream of the terminators in pHB1, pHB2, and pHB3 by the *Hin*dIII site, creating a set of *uidA* promoter‐probe vectors pHG1, pHG2, and pHG3, respectively. The complete nucleotide sequence of pHG3 (accession number: MK836323) has been deposited in GenBank.

The Xoo *hrpB1* and *hrpG* as well as the Xoc *hrpG*_xoc_ and *hrpX*_oc_ promoter regions were cloned into pNG1 to create the *hrp* promoter‐*uidA* fusions. The recombinants were cloned into pHB1, pHB2, or pHB3 via *Hin*dIII, resulting in pHG3‐*hrpB1*pro, pHG2‐*hrpG*pro, pHG2‐*hrpG*_xoc_pro, and pHG2‐*hrpX*_oc_pro.

### 4.3 Western blot and RT‐qPCR assays

The western blotting assays in XOM3 were performed as our previous study (Xu et al., 2019). The RT‐qPCR assays in XOM3 were performed as our previous protocols (Li et al., 2020). The primers for *hrpG* and *rsmA* genes in the RT‐qPCR analysis are listed in Table S2. The *gyrB* or *rpoD* genes were used for normalization.

### 4.4 GUS activity assays

The quantitative GUS analyses of the Xoo and Xoc strains in XOM3, rice tissues, and tobacco tissues were performed following our previous protocols (Li et al., 2020). The UV absorbance values were measured by a fluorescence spectrophotometer (GloMax Multi Detection System, Promega). The GUS activity was calculated according to the standard curve formula *x* = (measured UV absorbance + 23,751)/5,942, where *x* is the enzyme activity value. The unit of the enzyme activity is 4‐methyl‐umbelliferyl β‐D‐glucuronide  nmol⋅mg^−1^⋅min^−1^.

The qualitative GUS analyses of the Xoo and Xoc strains in rice and tobacco tissues were performed as a modified protocol. Briefly, the infected leaf samples were immersed in GUS staining solution (50 mM phosphate buffer [pH 7.2], 2 mM K_3_Fe(CN)_6_, 2 mM K_4_Fe(CN)_6_, 10 mM EDTA [pH 8.0], 2 mM X‐gluc, 0.1% Triton X‐100) and incubated in dark conditions at 37 °C for 12–24 hr. The leaves were then soaked with 70% ethanol for almost 24 hr until they were completely bleached and then they were photographed. The staining of rice leaves was carried out in 10‐ml tubes while for tobacco leaves it was carried out in a 24‐well plate. In this study, each bacterial strain was replicated on three leaves, with three injection sites.

### 4.5 Bacterial growth curve

The bacterial populations in rice and tobacco tissues were analysed by a modified method. Briefly, 100 mg of infected tissues were collected at different time points in 2‐ml microfuge tubes, then the leaves were disinfected in 70% ethanol for 1 min and washed with sterilized water at least three times. The leaves were ground with a fully automatic rapid tissue grinder (JX‐FSTPRP) at a frequency of 55 Hz, twice for 15 s, then were kept at room temperature for 30 min. The solutions were then diluted (10^−1^ to 10^−5^) and plated on NA medium with appropriate antibiotics and cultured at 28 °C for 3 days. The number of colonies was counted and statistically analysed. The bacterial populations in rice leaves at 24 and 48 hpi, and in tobacco leaves at 2 hpi were used for the quantitative GUS analyses.

### 4.6 Pathogenicity assays

The Xoo and Xoc strains were prepared as described above. The suspensions were adjusted to an optical density (OD_600nm_) of 1, then were inoculated into 2‐week‐old rice leaves using a needleless syringe, and were sprayed on 4‐week‐old rice leaves for the pathogen tracing assay. The water‐soaked symptoms were observed and photographed at 3 or 5 dpi, and the symptoms of bacterial leaf blight were photographed at 15 dpi. The pathogenicity assays were performed independently at least twice and similar results were acquired.

## CONFLICT OF INTEREST

The authors declare that they have no competing interests.

## Supporting information


**FIGURE S1** Applications of the protein modular assembly system in mutant complementation. (a) Lesion lengths caused by the Xoc *rsmA* mutant RΔ*rsmA* and its complementary strains. (b) Expression of the relevant *hrpG*‐FLAG and *hrpG*‐3Myc in trans was capable of restoring pathogenicity on host rice in the *hrpG* mutant. Error bars indicate standard deviation (*SD*). Asterisks indicate statistically significant differences (mean ± *SD*, *n* = 3, **p ≤ .01). The *hrpG* native promoter was cloned in the *hrpG*‐FLAG fusion in the constructs of pH1‐*hrpG*‐FLAG/3Myc, pH2‐*hrpG*‐FLAG/3Myc, and pHM1‐*hrpG*‐FLAG/3Myc, whereas the *hrpG*‐FLAG fusions were driven by the constitutive *lac* promoter in the constructs of pH1plac‐*hrpG*‐FLAG/3Myc, pH2plac‐*hrpG*‐FLAG/3Myc, and pHM1plac‐*hrpG*‐FLAG/3Myc

**FIGURE S2** (a) The bacterial growth curves of PXO99^A^ and PΔ*hrpG* in host rice tissues. (b) Quantification of *hrpB1* expression of three *pilN* mutants in *hrp*‐inducing medium XOM3. (c) The bacterial growth curves of PXO99^A^ and PΔ*hrpG* in nonhost tobacco tissues. 2‐10, 8‐28, and 26‐30 are Tn*5* insertion mutants of the *pilN* gene in the PXO99^A^ background

**TABLE S1** Bacterial strains and plasmids used in this study

**TABLE S2** Primer sequences used in this study

## Data Availability

The data that support the findings of this study are available from the corresponding author upon reasonable request.
