# Supplementary figures and images for: An improved, versatile and efficient modular plasmid assembly system for expression analyses of genes in Xanthomonas oryzae
Source: Mol Plant Pathol. 2021 Jan 24;22(4):480–92. doi: 10.1111/mpp.13033 (PMC7938625; doi:10.1111/mpp.13033)

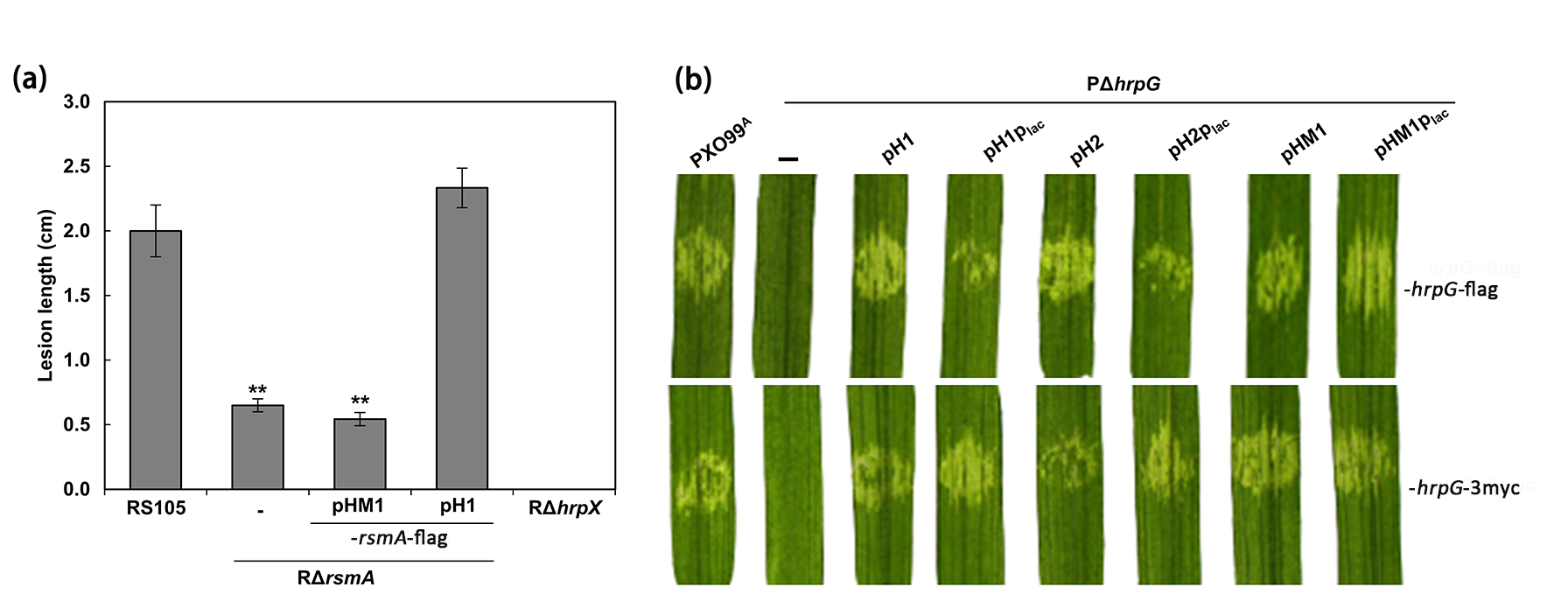

Supplement: Supplementary file 1 — FIGURE S1 Applications of the protein modular assembly system in mutant complementation. (a) Lesion lengths caused by the Xoc rsmA mutant RΔrsmA and its complementary strains. (b) Expression of the relevant hrpG‐FLAG and hrpG‐3Myc in trans was capable of restoring pathogenicity on host rice in the hrpG mutant. Error bars indicate standard deviation (SD). Asterisks indicate statistically significant differences (mean ± SD, n = 3, **p ≤ .01). The hrpG native promoter was cloned in the hrpG‐FLAG fusion in the constructs of pH1‐hrpG‐FLAG/3Myc, pH2‐hrpG‐FLAG/3Myc, and pHM1‐hrpG‐FLAG/3Myc, whereas the hrpG‐FLAG fusions were driven by the constitutive lac promoter in the constructs of pH1plac‐hrpG‐FLAG/3Myc, pH2plac‐hrpG‐FLAG/3Myc, and pHM1plac‐hrpG‐FLAG/3Myc [file MPP-22-480-s001.tif]

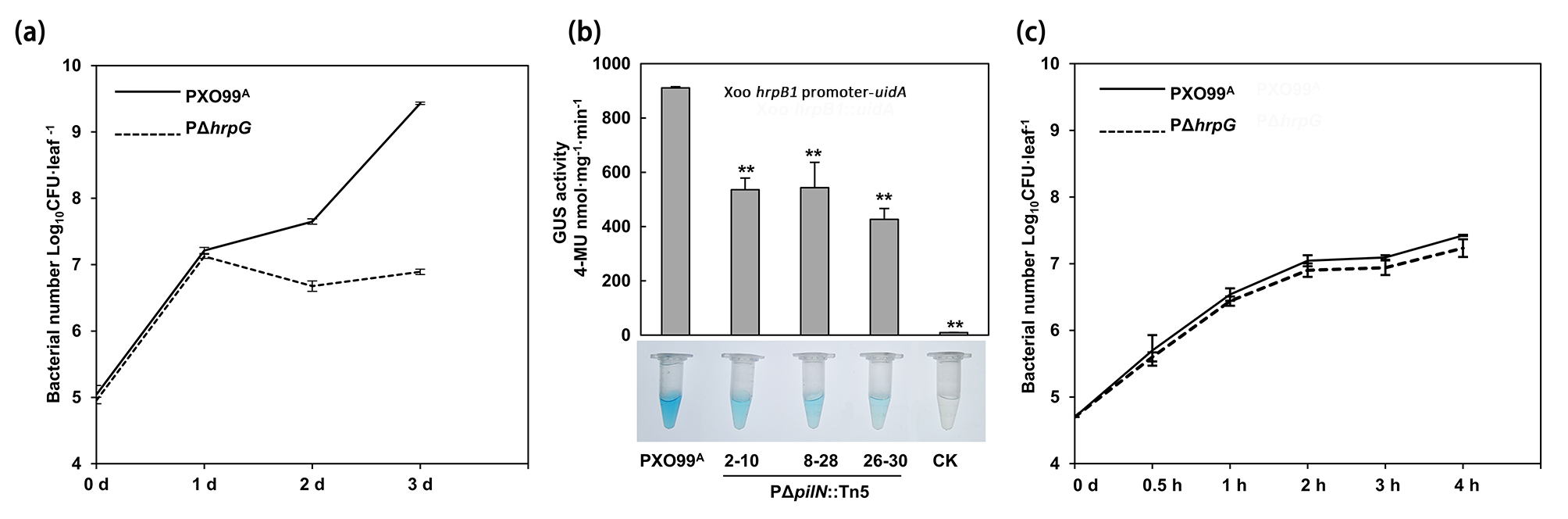

Supplement: Supplementary file 2 — FIGURE S2 (a) The bacterial growth curves of PXO99A and PΔhrpG in host rice tissues. (b) Quantification of hrpB1 expression of three pilN mutants in hrp‐inducing medium XOM3. (c) The bacterial growth curves of PXO99A and PΔhrpG in nonhost tobacco tissues. 2‐10, 8‐28, and 26‐30 are Tn5 insertion mutants of the pilN gene in the PXO99A background [file MPP-22-480-s004.tif]
