# Supplementary material for: An improved, versatile and efficient modular plasmid assembly system for expression analyses of genes in Xanthomonas oryzae
Source: Mol Plant Pathol. 2021 Jan 24;22(4):480–92. doi: 10.1111/mpp.13033 (PMC7938625; doi:10.1111/mpp.13033)
Supplement: Supplementary file 3 — TABLE S1 Bacterial strains and plasmids used in this study [file MPP-22-480-s002.docx]

# Supplementary Table S1. Bacterial strains and plasmids used in this study

| **Strains or plasmids** | | Relevant characteristics^a^ | Source |
| --- | --- | --- | --- |
| **Strains** | | | |
| *Xanthomonas* *oryzae* pv.*oryzae* | | | |
| PXO99^A^ | The *Xoo* wild-type strain, Philippine race 6 | | ([Hopkins *et a*l., 1992](#_ENREF_18)) |
| PΔ*hrpG* | *hrpG* deletion mutant of PXO99^A^ | | This laboratory |
| PΔ*hrpX* | *hrpX* deletion mutant of PXO99^A^ | | This laboratory |
| PΔ*trh* | *trh* insertion mutant of PXO99^A^; Km^r^ | | This laboratory |
| 2-10 | *pilN* Tn5 insertion mutant of PXO99^A^; Km^r^ | | ([Li *et al*., 2020](#_ENREF_28)) |
| 8-28 | *pilN* Tn5 insertion mutant of PXO99^A^; Km^r^ | | ([Li *et al*., 2020](#_ENREF_28)) |
| 26-30 | *pilN* Tn5 insertion mutant of PXO99^A^; Km^r^ | | ([Li *et al*., 2020](#_ENREF_28)) |
| *Xanthomonas* *oryzae* pv. *oryzicola* | | |  |
| RS105 | The *Xoc* wild-type strain, Chinese race 2 | | ([Li *et al*., 2011](#_ENREF_29)) |
| RΔ*hrpG* | *hrpG* deletion mutant of RS105 | | ([Li *et al*., 2011](#_ENREF_29)) |
| RΔ*rsmA* | *rsmA* deletion mutant of RS105 | | ([Song *et al*., 2017](#_ENREF_37)) |
| RΔ*trh* | *trh* deletion mutant of RS105 | | ([Li *et al*., 2011](#_ENREF_29)) |
| *Escherichia coli* |  | |  |
| DH5a | φ901ac ZΔM15, recA1 | | Invitrogen |
| **Plasmids** |  | |  |
| pHM1 | A broad-host-range cloning vector, *mob*, pSa ori, *lac* P^+^ ; Sp^r^ | | ([Xu *et al*., 2019](#_ENREF_45)) |
| pH1 | The pHM1 derivative backbone vector harbouring the T_0_T_1_ terminators; Sp^r^ | | This study |
| pH2 | The pHM1 derivative backbone vector harbouring the (T_1_)_4_ terminators; Sp^r^ | | This study |
| pH3 | The pHM1 derivative backbone vector harbouring the (T_1_)_4_ and T_0_T_1_ terminators; Sp^r^ | | ([Xu *et al*., 2019](#_ENREF_45)) |
| pBluescript SK | A high-copy cloning vector with ColE1 origin of replication, Ap^r^ | | Strategene |
| pHB1 | The high-copy destination vector derived from pH1; Sp^r^, Ap^r^ | | This study |
| pHB2 | The high-copy destination vector derived from pH2; Sp^r^, Ap^r^ | | This study |
| pHB3 | The high-copy destination vector derived from pH3; Sp^r^, Ap^r^ | | This study |
| pSV-flag | The entry vector for protein expression; Ap^r^ | | ([Xu *et al*., 2019](#_ENREF_45)) |
| pSV-3myc | The entry vector for protein expression; Ap^r^ | | This study |
| pNG | The entry vector for promoter activity; km^r^ | | This study |
| pNG1 | The entry vector for promoter activity; Km^r^ | | This study |
| pHG1 | The *uidA* promoter-probe vector; Sp^r^ | | This study |
| pHG2 | The *uidA* promoter-probe vector; Sp^r^ | | This study |
| pHG3 | The *uidA* promoter-probe vector; Sp^r^ | | This study |
| pH1-flag | The protein expression vector; Sp^r^ | | This study |
| pH2-flag | The protein expression vector; Sp^r^ | | This study |
| pH3-flag | The protein expression vector; Sp^r^ | | ([Xu *et al*., 2019](#_ENREF_45)) |
| pH1P_lac_-flag | The protein expression vector for constitutive expression; Sp^r^ | | This study |
| pH2P_lac_-flag | The protein expression vector for constitutive expression; Sp^r^ | | This study |
| pH1-3myc | The protein expression vector; Sp^r^ | | This study |
| pH2-3myc | The protein expression vector; Sp^r^ | | This study |
| pH3-3myc | The protein expression vector; Sp^r^ | | This study |
| pH1P_lac_-3myc | The protein expression vector for constitutive expression; Sp^r^ | | This study |
| pH2P_lac_-3myc | The protein expression vector for constitutive expression; Sp^r^ | | This study |
| pH1-*hrpG*::flag | The fusion of Xoo *hrpG*-flag cloned in pH1; Sp^r^ | | This study |
| pH2-*hrpG*::flag | The fusion of Xoo *hrpG*-flag cloned in pH2; Sp^r^ | | This study |
| pHM1-*hrpG*::flag | The fusion of Xoo *hrpG*-flag cloned in pHM1; Sp^r^ | | This study |
| pH1p_lac_-*hrpG*::flag | The Xoo *hrpG*-flag fusion under control of the *lac* promoter in pH1; Sp^r^ | | This study |
| pH2p_lac_-*hrpG*::flag | The Xoo *hrpG*-flag fusion under control of the *lac* promoter in pH2; Sp^r^ | | This study |
| pHM1p_lac_-*hrpG*::flag | The Xoo *hrpG*-flag fusion under control of the *lac* promoter in pHM1; Sp^r^ | | This study |
| pH1-*hrpG*::3myc | The fusion of Xoo *hrpG*-3myc cloned in pH1; Sp^r^ | | This study |
| pH2-*hrpG*::3myc | The fusion of Xoo *hrpG*-3myc cloned in pH2; Sp^r^ | | This study |
| pH1-*hrpX*::flag | The fusion of Xoc *hrpX*-flag cloned in pH1; Sp^r^ | | This study |
| pHM1-*hrpX*::flag | The fusion of Xoc *hrpX*-flag cloned in pHM1; Sp^r^ | | This study |
| pHM1-*rsmA*::flag | The fusion of Xoc *rsmA*-flag cloned in pHM1; Sp^r^ | | ([Song *et al*., 2017](#_ENREF_37)) |
| pH1-*rsmA*::flag | The fusion of Xoc *rsmA*-flag cloned in pH1; Sp^r^ | | This study |
| pHG3-*hrpB1*pro | The Xoo *hrpB1* promoter and *uidA* fusion cloned in pH3; Sp^r^ | | This study |
| pHG2-*hrpG*pro | The Xoo *hrpG* promoter and *uidA* fusion cloned in pH2; Sp^r^ | | This study |
| pHG2-*hrpG_xoc_*pro | The Xoc *hrpG* promoter and *uidA* fusion cloned in pH2; Sp^r^ | | This study |
| pHG2-*hrpX_oc_*pro | The Xoc *hrpX* promoter and *uidA* fusion cloned in pH2; Sp^r^ | | This study |

^a^Ap^r^, ampicillin resistance; Km^r^, kanamycin resistance; Gm^r^, gentamycin resistance; Sp^r^, streptomycin resistance
