# Supplementary material for: An improved, versatile and efficient modular plasmid assembly system for expression analyses of genes in Xanthomonas oryzae
Source: Mol Plant Pathol. 2021 Jan 24;22(4):480–92. doi: 10.1111/mpp.13033 (PMC7938625; doi:10.1111/mpp.13033)
Supplement: Supplementary file 4 — TABLE S2 Primer sequences used in this study [file MPP-22-480-s003.docx]

**Supplementary Table S2**: Primer sequences used in this study

| Primers | Sequences (5’ to 3’) | Reference or source |
| --- | --- | --- |
| *gusA*-F(*Nde*I) | GGGCATATGATGTTACGTCCTGTAGAAAC | This study |
| *gusA*-R(*Nde*I) | CATCATATGTCATTGTTTGCCTCCCTGCT | This study |
| *gusA*-F | ACTGCCAGGAATTGGGGATCGGAAG | This study |
| *gusA*-R | CATAAGCTTTCATTGTTTGCCTCCCTGCT | This study |
| T01-F | AGAGAATTCGACTCCTGTTGATAGATCCAG | This study |
| T01-R | AGTAAGCTTGGCGGATTTGTCCTACTCAG | This study |
| T14-F | GGATCCTGTTGGGAAGGGCGATCGGTGCGGGCCTCT | ([Xu *et al*., 2019](#_ENREF_45)) |
| T14-F | GGCATGCAAGCTTCCGATCCCCAATTCCTG | ([Xu *et al*., 2019](#_ENREF_45)) |
| *hrpG*-F | GGAGTCGACGCAGGTAGTCGGACAACGTCT | ([Xu *et al*., 2019](#_ENREF_45)) |
| *hrpG* ORF-F | GGAGTCGACATGAACGACCCTTGCCCCCTTGC | This study |
| *hrpG*-R | CATGGTACCGCAGGCGGCTGTGCGAT | ([Xu *et al*., 2019](#_ENREF_45)) |
| *hrpXoc*-F | AATGGATCCACATCCGCCAAGGTCTAGACCAAC | This study |
| *hrpXoc*-R | GTAGAATTCCCGTTGCAAGGTTTCCATCGG | This study |
| *hrpG* promoter-F | GGAGTCGACGCAGGTAGTCGGACAACG | ([Xu *et al*., 2019](#_ENREF_45)) |
| *hrpG* promoter -R | GAGGAATTCTGAAGGGGAGCGCAAGCACC | ([Xu *et al*., 2019](#_ENREF_45)) |
| *hrpB1* promoter-F | GGAGTCGACGCGACACCGGCTTGAGCGAG | ([Xu *et al*., 2019](#_ENREF_45)) |
| *hrpB1* promoter-R | GTGGAATTCCTTTCAGCTCTGGCCGCAGT | ([Xu *et al*., 2019](#_ENREF_45)) |
| *hrpG_xoc_* promoter -F | GGGGTCGACCCTGCTCGTATAGGTAGGAAGAC | This study |
| *hrpG*_xoc_ promoter -R | GTTGAATTCACACCGAGACATTGCGCGATA | This study |
| *hrpX_oc_* promoter -F | GAAGGATCCTTCGCAGACGTTGTCCGAAAG | This study |
| *hrpX*_oc_ promoter -R | TCTGGTACCGCATCGCCGCTGGCACCAAAC | This study |
| *rsmA* qRT-F | ACCTTGATGATCGGCGACTC | This study |
| *rsmA* qRT-R | TGATAGATTTCTTCACGATG | This study |
| *hrpG* qRT-F | GTTGCTCCGCGACGAAAATACCGTG | This study |
| *hrpG* qRT-R | CTGCTCCATGGTGCGGTCGGTGAAT | This study |
| *rpoD* qRT-F | CGACAACACCACCAACATCAATC | ([Li *et al*., 2020](#_ENREF_28)) |
| *rpoD* qRT-R | GCTTACCGACCTCTTCCAACG | ([Li *et al*., 2020](#_ENREF_28)) |
| *gyrB* qRT-F | CGGCACTTACGACTCCAGCAAG | ([Li *et al*., 2020](#_ENREF_28)) |
| *gyrB* qRT-R | CGACCAGGATTTTCACCACGATG | ([Li *et al*., 2020](#_ENREF_28)) |
